# Influence of MyD88 and αβ T cells on mesenteric lymph node innate lymphoid cell populations during *Toxoplasma gondii* infection

**DOI:** 10.1371/journal.pone.0322116

**Published:** 2025-04-29

**Authors:** Jessica Belmares-Ortega, Fatouma Zara Issoufou Kapran, Eric Y. Denkers

**Affiliations:** Center for Evolutionary and Theoretical Immunology and Department of Biology, University of New Mexico, Albuquerque, New Mexico, United States of America; The University of Texas MD Anderson Cancer Center, UNITED STATES OF AMERICA

## Abstract

First encounter of *Toxoplasma* with the host immune system occurs within tissues of the intestine, including the intestinal mucosa and draining lymph nodes. In this study, we focused on the mesenteric lymph node compartment, the central hub of adaptive immune induction following orally acquired infection. We examined innate lymphoid cells (ILC) in mesenteric lymph nodes during *Toxoplasma* infection, determining the influence of MyD88 and the T lymphocyte compartment on ILC subset distribution, IFN-γ production, MHC class II expression and proliferation. Collectively, we observed an ILC1-dominated response that was impacted by both MyD88 and T lymphocytes. We also found a population of putative ILC that were negative for signature transcription factors associated with ILC1, 2 and 3 subsets. This study increases our understanding of ILC-mediated immunity during *Toxoplasma* infection and points to the complex interactions with which these cells engage T cell and MyD88-dependent immunity.

## Introduction

*Toxoplasma gondii* is a highly prevalent parasitic pathogen that can cause significant health issues in immunocompromised individuals and following congenital infection [1,2]. The parasite infects humans, domestic animals and wildlife throughout the world. In cats, *Toxoplasma* undergoes sexual reproduction resulting in fecal shedding of oocysts into the environment. The parasite can then be transmitted through ingestion of contaminated food or water, initiating infection in the gastrointestinal tract [3]. Also contributing to the great success of this parasite, *Toxoplasma* is capable of oral transmission from one host to another through direct carnivorism. Following entry into the intestinal tract, the parasite crosses the epithelial barrier and migrates through the lymphatic system leading to widespread dissemination and the establishment of chronic infection. The latter is characterized by formation of long-lived tissue cysts, particularly in the brain and muscle. In immunodeficient populations, such as HIV/AIDS patients, the parasite may reactivate during chronic infection, resulting in life-threatening tissue destruction and inflammation [1].

Immune recognition and initiation of innate and adaptive immunity first occurs within the lamina propria (LP) of the small intestine [4]. A robust interferon-gamma (IFN-γ) response is critical for host protection, and while the predominant source of this cytokine is CD4^+^ and CD8^+^ T lymphocytes, NK cells and neutrophils are also capable of supplying this cytokine, particularly during initial stages of *T. gondii* infection [5–7]. The MyD88 signaling pathway plays an important role in orchestrating this response in mice, largely through induction of the cytokine IL-12 that fuels emergence of strong Type I cytokine-based immunity [8–10]. A predominant part of the MyD88-driven response is triggered by TLR11/12 recognition of the parasite protein profilin during early stages of infection [11–13]. Other TLRs, such as TLR2, TLR4, and TLR9, have also been implicated in recognition of *Toxoplasma* [14]. Although MyD88-dependent pathways are important, it is also clear that MyD88-independent mechanisms of immunity contribute to optimal IL-12 and IFN-γ production during *Toxoplasma* infection [15–17]. In this regard, parasite molecules such as GRA24 and GRA15_II_ are known to trigger IL-12 through MyD88-independent signaling mechanisms that bypass this adaptor molecule and directly activate NFκB and mitogen-activated protein kinase signal transduction [18–20].

Innate lymphoid cells (ILC) are innate immune cells derived from the common lymphoid precursor that also gives rise to the T and B cell lineages. There are five currently recognized types of ILC including lymphoid tissue-inducer (LTi) cells, natural killer (NK) cells, ILC1, ILC2 and ILC3 cells [21,22]. LTi cells are involved in development of secondary lymphoid tissues and NK cells are known for cytotoxic activity against tumors and virally infected cells. ILC1, ILC2, and ILC3 are regarded as innate counterparts to Th1, Th2 and Th17 lymphocytes. There is also evidence for an additional ILC type that possesses regulatory function, therefore mirroring the Treg T cell subset [23,24]. ILC subsets are known to interconvert in response to environmental cues and cytokine signaling [25]. This plasticity allows ILC to adapt to diverse immune challenges [26]. A wide range of immunomodulatory receptors, including major histocompatibility (MHC) class II proteins, are expressed by ILC, endowing these cells with the ability to modulate innate and adaptive immunity [27–29].

ILC3 are abundant in the intestinal LP, whereas the ILC1 group dominates in the mesenteric lymph nodes (MLN) [30]. ILC have been recognized in providing protection against a variety of pathogens, including *Salmonella typhimurium*, *Helicobacter pylori,* and *Toxoplasma* [26,31]. In *Toxoplasma* infection, ILC1 have been shown to be important for IFN-γ-dependent host resistance and maintenance of IRF8^+^ inflammatory dendritic cells [32,33]. It has also been found that infection with *T. gondii* triggers conversion of NK cells to an ILC1-like cell type [34]. Recent data from our laboratory indicate that in the small intestinal LP, both ILC1 and ILC3 produce IFN-γ, though only ILC1 rely on the Toll-like receptor (TLR)/IL-1R adaptor MyD88 for optimal function. The microbiota also contributes to shaping ILC responses, particularly in regulating ILC1-derived IFN-γ production during *Toxoplasma* infection [15].

In this study we investigate ILC populations in the MLN following orally initiated infection with *Toxoplasma*. This secondary lymphoid tissue serves as an information hub organizing interactions between the T lymphocyte compartment, innate immunity and pathogen-derived antigens. Here, we determine how MyD88 influences ILC distribution and function during *T. gondii* infection, and we examine the impact of T lymphocytes themselves on ILC activity in the MLN.

## Results

### Distinct ILC populations are present in the MLN following *T. gondii* infection

**Fig 1:**
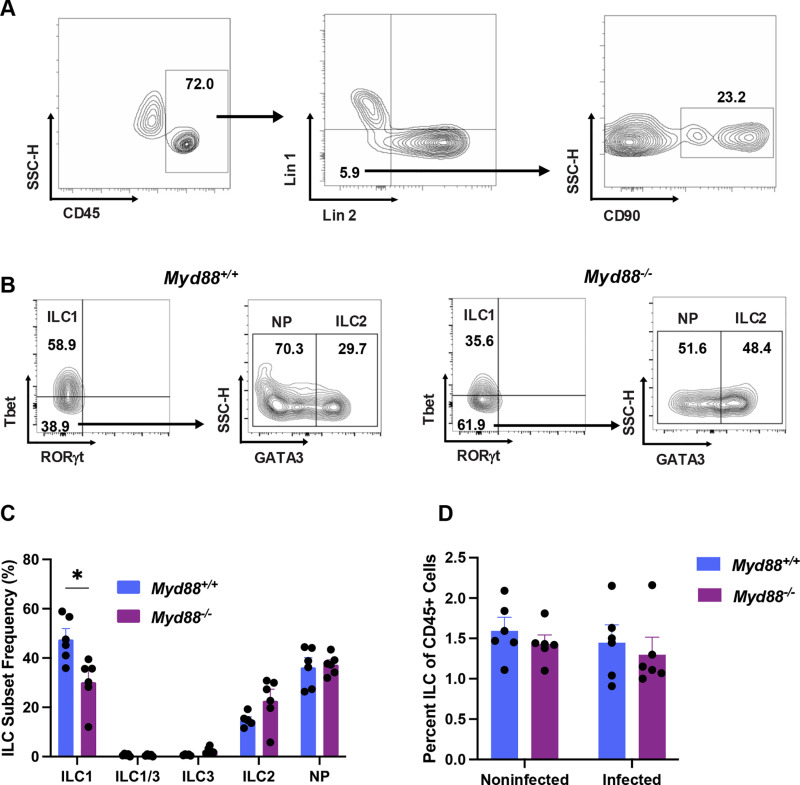
Distribution of ILC subsets in the MLN during acute *T. gondii* infection. MLN cells were collected 10 days post oral infection from *MyD88*^*+/+*^ and *MyD88*^*-/-*^ mice. (A) Representative flow plots of *MyD88*^*+/+*^ cells defining ILC as CD45^+^(left scatter plot), Lineage-1^-^ (CD5, CD8α, CD3ε) and Lineage-2^-^ (B220, CD11c, CD11b) (middle scatter plot), and CD90^+^ (right scatter plot). One representative wild-type mouse is shown. (B) ILC subset identification in WT and MyD88 KO mice based upon expression of T-bet and RORγt (ILC1, T-bet^+^RORγt^-^; ILC3, T-bet^-^RORγt^+^; ILC1/3, T-bet^+^RORγt^+^). Gating on T-bet^-^RORγt^-^ and examination of GATA3 expression defines a population of ILC2 (T-bet^-^RORγt^-^GATA3^+^) and an undefined population of T-bet^-^RORγt^-^GATA3^-^ cells (NP, null population). One representative wild-type and one representative knockout mouse is shown. (C) ILC subset distribution in infected *MyD88*^*+/+*^ and *MyD88*^*-/-*^ mice where each symbol represents an individual animal. The graph shows ILC subset frequency relative to total ILC. (D) Percentage ILC out of total CD45^+^ MLN cells in noninfected versus Day 10-infected mice. In the scattergrams, numbers indicate percentages falling within the indicated quadrants. Data shown in (C) and (D) are SEM of one of two independent experiments. Unpaired Student’s t test *, p < 0.05.

We previously characterized the influence of MyD88 on ILC populations of the LP and peritoneal cavity during infection with *T. gondii* [15,16]. Here, we extend our analysis to the MLN compartment, a major site of acquired immunity during intestinal infection. Wild-type (WT) and MyD88 knockout (KO) mice were orally inoculated with cysts of the Type II *Toxoplasma* strain ME49, then 10 days later MLN cells were collected for multi-color flow cytometric analysis. Following dead and doublet cell exclusion, ILC were identified amongst CD45^+^ cells based upon gating for Lineage-1^-^ (CD5^-^, CD8α^-^, CD3ε^-^), and Lineage-2^-^ (B220^-^, CD11c^-^, CD11b^-^) cells, followed by gating for CD90^+^ cells (**Fig 1A**). To identify putative ILC1 and ILC3 subsets amongst Lin1^-^Lin2^-^CD90^+^ cells, expression of T-bet and RORγt was examined. Amongst T-bet^-^RORγt^-^ cells, ILC2 were identified based upon expression of GATA3 (**Fig 1B**).

The most predominant ILC during infection was the ILC1 subset. In the absence of MyD88 there was a partial but significant decrease in their frequency (Fig 1B and C). There was also a population of cells that expressed none of the classical markers of ILC1/2/3 whose identity is unclear (null population, NP). The ILC2 subset was also present and was unaffected by presence or absence of MyD88. ILC3 cells and a population of ILC co-expressing T-bet and RORγt (designated ILC1/3), a subset we previously identified in the peritoneal cavity were not detected in the MLN (Fig 1C). Because the absence of ILC3 was unexpected, we confirmed that our antibody and staining protocol were effective. As shown in S1 Fig, we observed robust RORγt expression by CD4^+^CD8^+^ double-positive thymocytes from noninfected mice whereas this ILC3/Th17 transcription factor was absent in the CD4^+^ single-positive population. This result was reported by others, and it confirms the effectiveness of our antibody staining protocol [35].

To determine how *Toxoplasma* influenced the ILC population as a whole, we compared total ILC from noninfected and infected mice. As shown in Fig 1D, infection did not influence the overall frequency of ILC in either *Myd88*^*+/+*^ or *Myd88*^*-/-*^ mice. To further determine the steady-state distribution of ILC subsets, we examined MLN, LP, and splenic tissues from noninfected WT mice (S2 Fig). The ILC subset distribution in non-infected MLN overall closely resembled the pattern in infected MLN, with a predominance of ILC1 (S2A Fig and S2B Fig, Fig 1C). In noninfected LP, the subsets were more evenly distributed, ranging from approximately 10–30% (S2C Fig and S2D Fig). The ILC subset distribution in noninfected spleen displayed a similar pattern to that found in tissues of the MLN (S2E Fig and S2F Fig). We conclude that presence of ILC1 is partially dependent upon MyD88, and that *T. gondii* infection does not alter the overall frequency of total ILC or the distribution of ILC subsets.

### Influence of MyD88 on ILC IFN-γ expression

**Fig 2:**
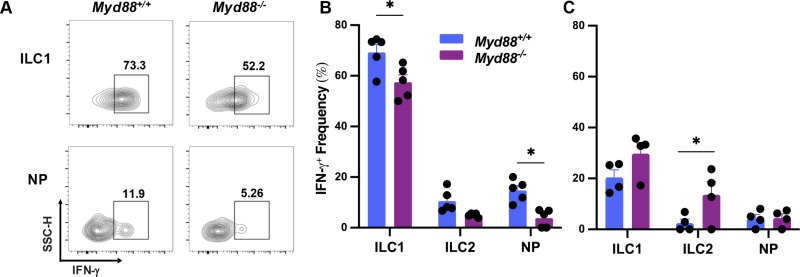
IFN- γ expression by ILC1 and NP is partially impaired in the absence of MyD88. Expression of IFN-γ by ILC isolated from MLN of Day 10-orally infected mice was analyzed by flow cytometry. (A) Representative scatter plots showing IFN-γ expression in ILC1 and NP populations from WT and MyD88 KO mice. Gating for ILC1 and NP cells was accomplished as described in Fig 1. The numbers in each quadrant indicate percentages relative to total ILC1 or NP cells. (B) Overall IFN-γ expression levels, where each symbol represents a single mouse. (C) Expression of IFN-γ by ILC isolated from MLN of noninfected mice. Data shown are SEM of one of two independent experiments. Each symbol represents a single mouse. Unpaired Student’s t test was used for statistical analysis, * p < 0.05.

We and others previously found that peritoneal cavity and LP ILC1 are a source of IFN-γ during *Toxoplasma* infection [15,16,32,34,36]. Consistent with these results, we also found that MLN ILC1 possess the capacity to produce IFN-γ during infection (**Fig 2A**, left scatterplots and **Fig 2B**, blue-shaded bars). ILC2 and T-bet^-^RORγt^-^GATA3^-^ (NP) ILC produce markedly less IFN-γ. To determine the extent to which MyD88 is involved in IFN-γ expression by MLN ILC, these experiments were carried out in parallel with *Myd88*^*-/-*^ mice (**Fig 2A**, right scattergrams, **Fig 2B**, magenta-shaded bars). We found a marginal decrease in ILC1 IFN-γ expression in the absence of MyD88 (approximately 20%). While NP cell IFN-γ expression was lower compared to ILC1, absence of MyD88 had a greater impact (approximately 70% reduction; **Fig 2A**, lower scatterplots and **Fig 2B**). To determine the extent to which IFN-γ expression was infection-driven, we also examined cytokine expression in noninfected mice (**Fig 2C**). For ILC1, we found lower IFN-γ expression in WT and KO mice. Expression of IFN-γ by ILC2 and NP cells overall remained low in noninfected mice. Overall, these results are in line with our previous findings focused on LP ILC, where MyD88 had only a minor impact on ILC1 IFN-γ expression [16]. They contrast with ILC1 in the peritoneal cavity, where the absence of MyD88 had a much more profound impact on IFN-γ production by ILC1 [15].

### Functional activity of ILC1 is regulated by IL-12

**Fig 3:**
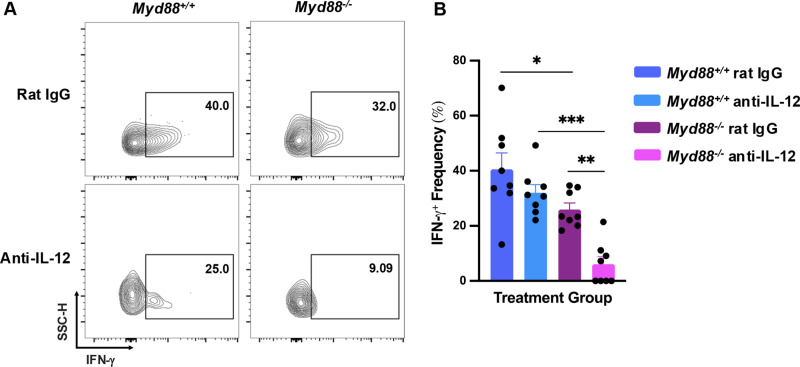
Expression of IFN- γ in ILC1 is highly IL-12 dependent in the absence of MyD88. Mice were administered anti-IL-12p40 mAb or control rat IgG before oral inoculation and continuing until 10 days post infection when MLN were harvested. IFN-γ expression frequency was analyzed by flow cytometry. (A) Representative flow plots of ILC1 IFN-γ expression. Gating for ILC1 was accomplished as in Fig 1. Numbers associated with each quadrant indicate the percent ILC1 positive for IFN-γ. (B) Collective results obtained from two independent experiments. Error bars indicate SEM values. Each symbol represents a single mouse. One-way ANOVA with Tukey’s multiple comparison test was used for analysis where * p < 0.05, ** p < 0.01, and *** p < 0.001.

The cytokine IL-12 is known to regulate ILC IFN-γ production [15,37,38], and we previously found that this cytokine impacts ILC1 proliferation in the peritoneal cavity. Therefore, we employed a monoclonal antibody (mAb) depletion approach to determine how IL-12 influences ILC1 expression of IFN-γ in the presence and absence of MyD88. In *MyD88*^*+/+*^ mice, ILC1 IFN-γ expression trended lower in IL-12-depleted mice (**Fig 3A**, left scatter plots), but this effect was not statistically significant over multiple animals (**Fig 3B**, blue-shaded bars). Interestingly, IFN-γ that was expressed independently of MyD88 was highly reliant on IL-12, showing an overall 80% decrease in expression following mAb treatment (**Fig 3A**, right scatter plots, and **Fig 3B**, magenta shaded bars). Thus, IFN-γ expression by ILC1 consists of both MyD88-dependent and MyD88-independent components, and the component not requiring MyD88 is highly reliant upon IL-12.

### ILC expression of MHC class II during *T. gondii* infection

**Fig. 4:**
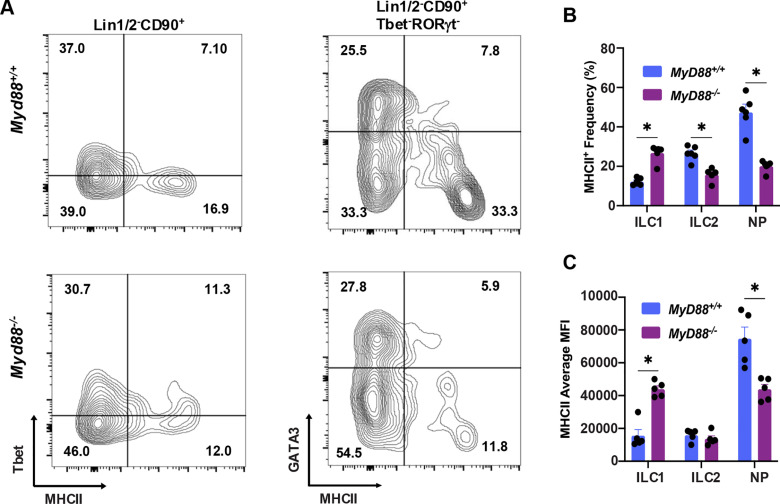
ILC MHC class II expression is partially controlled by MyD88. ILC populations in MLN cells from orally inoculated mice were analyzed by flow cytometry for MHC class II expression. (A) Left scatter plot, T-bet vs. MHC class II staining on Lin1/2^-^CD90^+^ ILC. Right scatter plot, GATA3 vs. MHC class II expression on Lin1/2^-^CD90^+^T-bet^-^RORγt^-^ cells. The numbers associated with each quadrant indicate percentages relative to the four quadrants together. One representative wild-type and one representative knockout mouse is shown. (B) Frequency of MHC class II-expressing ILC1, ILC2 and NP cells relative to each respective subset. (C) Mean fluorescence intensity (MFI) of class II expression on ILC1, ILC2 and NP cells. Each symbol represents a single mouse. Data shown in (B) and (C) are mean and SEM of one of two independent experiments. Each symbol represents a single mouse (n=5/group). Unpaired Student’s t test was used to assess statistical significance where * p < 0.05.

It has been reported that ILC, in particular ILC2 and ILC3, can express MHC class II molecules and therefore potentially exert a direct influence on antigen-specific T cell function through cell-cell interaction [28,29]. Therefore, we assessed MHC expression on ILC1, ILC2 and NP cells in the MLN during infection. Amongst WT Lin1/2^-^CD90^+^T-bet^+^ cells (ILC1), approximately 10% expressed MHC class II protein (**Fig 4A**, top left scatterplot, and **Fig 4B**). Higher levels were expressed by WT Lin1/2^-^CD90^+^T-bet^-^RORγt^-^GATA3^+^ (ILC2; approximately 30%). Lin1/2^-^CD90^+^T-bet^-^RORγt^-^GATA3^-^ (NP) cells expressed the highest levels of MHC class II protein with on average 50% staining positive (**Fig 4A**, top right scatterplot and **Fig 4B**). Interestingly, in MyD88 KO mice, class II MHC expression was increased in ILC1 but there was a concomitant decrease in NP and to a lesser extent ILC2 populations (**Fig 4A**, lower scatterplots and **Fig 4B**). Along similar lines, mean fluorescence intensity (MFI) of MHC class II-positive cells in ILC1 was significantly increased in the absence of MyD88 (**Fig 4C**). In the NP population absence of this signaling adaptor decreased class II MFI in *MyD88*^*-/-*^ mice (**Fig 4C**).

### MyD88 positively regulates MLN ILC proliferation

**Fig 5:**
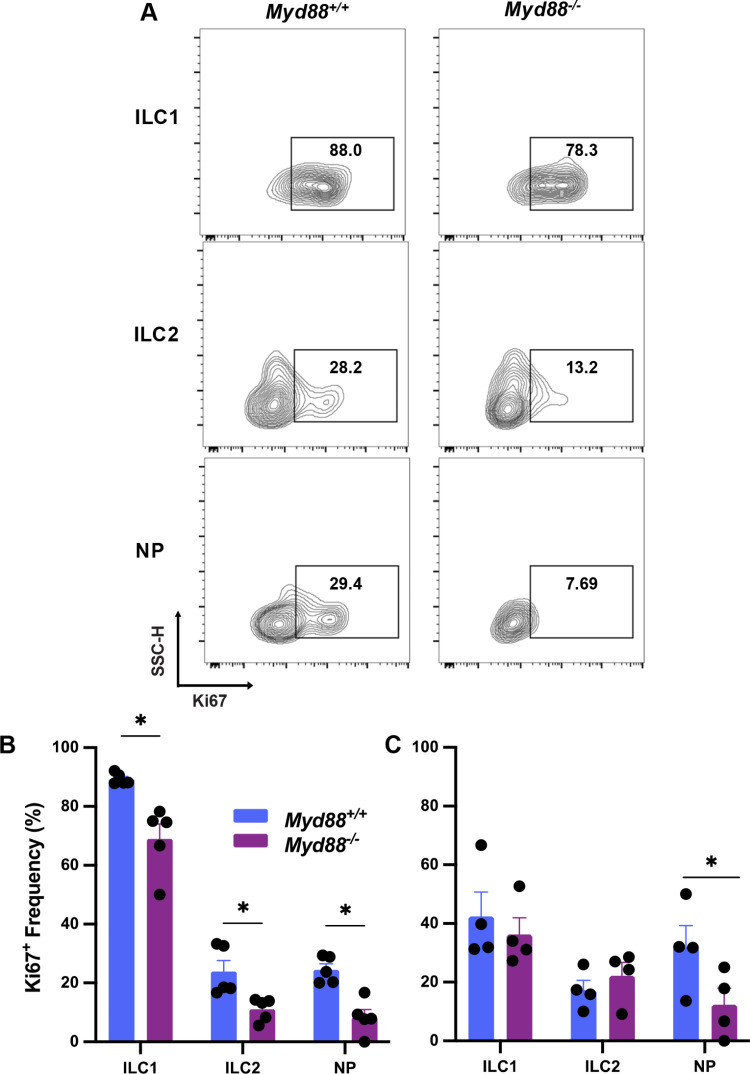
ILC proliferation is in part promoted by MyD88. (**A**) Expression of Ki67 in MLN ILC1, ILC2 and NP populations of MyD88^+/+^ and MyD88^-/-^ mice. Cells were collected 10 days post oral infection and subsequently analyzed by flow cytometry. (**B**) Frequency of Ki67^+^ ILC expressed as mean percentage over multiple mice. (**C**) Overall expression of Ki67 in ILC from noninfected mice. Data shown are mean ± SEM of one of two independent experiments. Each symbol represents a single mouse. Unpaired Student's t test was used to assess statistical significance where * p < 0.05.

We previously found high levels of ILC1 proliferation in the peritoneal cavity but not the LP compartment following respective i. p. and oral infection, as measured by expression of Ki67 [15]. As shown in Fig 5A and Fig 5B, we found that nearly 90% of *MyD88*^*+/+*^ MLN ILC1 expressed Ki67, and this number decreased by approximately 20% in MyD88 KO cells. Both ILC2 and NP cells expressed Ki67, although not to the same extent as ILC1. Loss of MyD88 also negatively impacted the proliferative capacity of ILC2 and NP cells (Fig 5A and B). In Fig 5C, we assessed proliferation in ILC from noninfected animals. For ILC1, Ki67 expression dropped from approximately 80% to 40%. Expression of Ki67 was largely unaffected by status of infection. The partial dependence of MLN ILC proliferation on MyD88 expression during infection contrasts to our previous study where we found no influence of this adaptor molecule on proliferation in peritoneal cavity ILC. It is possible that the influence of MyD88 on MLN ILC reflects the proximity of this tissue to the intestine harboring a large microbiota population.

### αβ T cell receptor-positive T cells influence MLN ILC frequency and function

**Fig 6:**
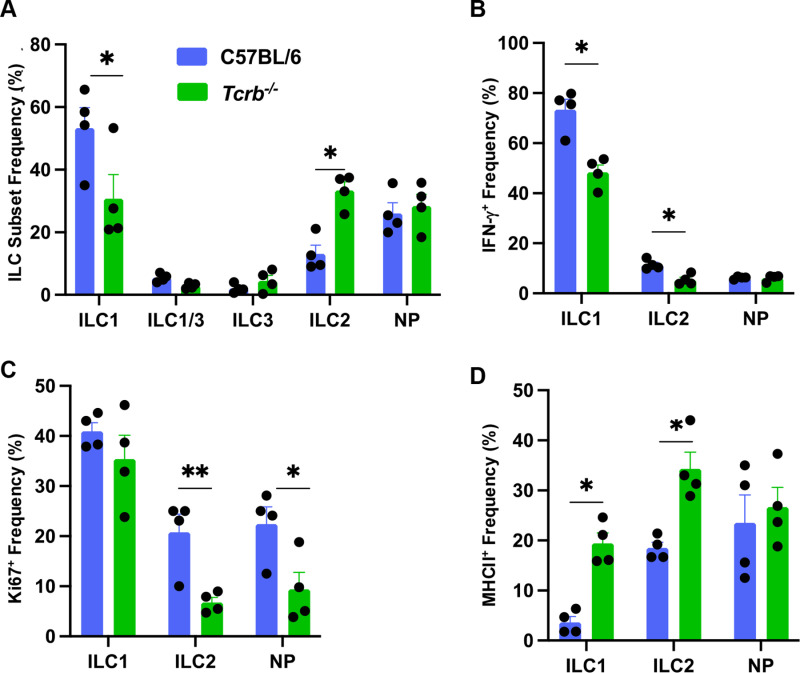
Impact of **αβ** **TCR-positive cells on ILC distribution and function.** (A) MLN from C57BL/6 and *Tcrb*^*-/-*^ from Day 10-infected mice were subject to flow cytometric staining and ILC subset distribution was determined. (B) Percent of each ILC subset expressing IFN-γ. Data shown are mean ± SEM of one of two independent experiments. (C) ILC frequency of Ki67 expression. Each symbol represents a single mouse. An unpaired Student’s t test was used to assess statistical significance where * p < 0.05.

While ILC are known to support T cell functions [27], we investigated whether αβ T cells, in turn, influence ILC populations. We analyzed ILC subset frequency (**Fig 6A**), IFN-γ expression (**Fig 6B**), proliferative capacity (**Fig 6C**) and MHC class II expression (**Fig 6D**) in WT (C57BL6/J) and αβ T cell receptor-negative *Tcrb*^*-/-*^ mice in Day 10 orally inoculated mice. As shown in **Fig 6A**, in mice lacking αβ T cells there was an approximately 50% decrease in ILC1 frequency accompanied by a concomitant 70% increase in ILC2 frequency. Capacity for IFN-γ production was similarly decreased in ILC1 cells isolated from mice lacking αβ T lymphocytes (**Fig 6B**). The ILC1 proliferative capacity, as measured by Ki67 expression (**Fig 6C**), was unaffected in *Tcrb*^*-/-*^ mice. Nevertheless, absence of αβ T cells decreased Ki67 expression in both ILC2 and NP subsets. Interestingly, both ILC1 and ILC2 displayed striking increases in expression of MHC class II in the absence of αβ T lymphocytes (**Fig 6D**). These results suggest that ILC operate in partial dependence on T cells during *Toxoplasma* infection, at least as measured by these parameters.

### MyD88 and αβ T cells influence MLN IFN-γ production

**Fig 7:**
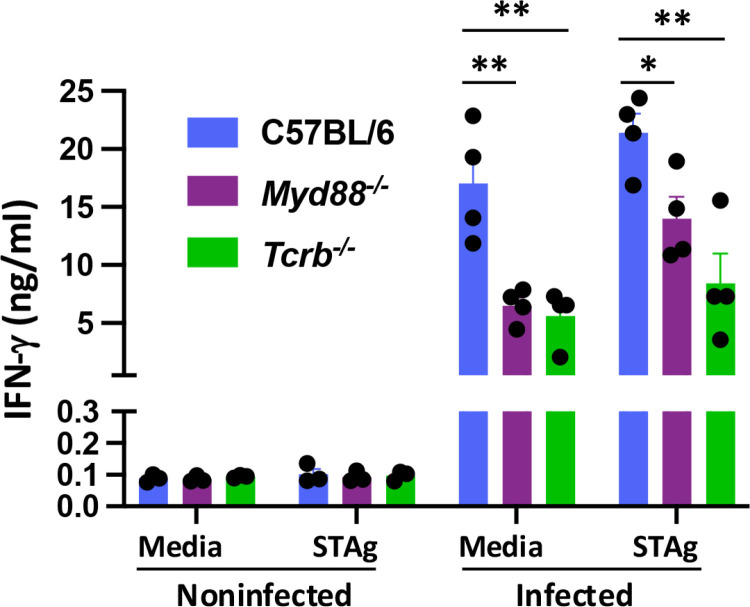
MLN IFN- **γ** **production is decreased in the absence of MyD88 and** **αβ** **T cells.** MLN cells of C57BL/6, *MyD88*^*-/-*^, and *Tcrb*^*-/-*^ mice were collected from noninfected mice or 10 days post oral infection, and incubated in media or STAg. Supernatants were collected 3 days later and IFN-γ was measured by ELISA. Data shown are mean ± SEM of one of two independent experiments. Each symbol represents a single mouse. One-way ANOVA with Tukey’s multiple comparison test was used to assess statistical significance, where * p < 0.05, ** p < 0.01, *** p < 0.001.

Lastly, to gain an overall view of the influence of T cells and MyD88 in MLN IFN-γ secretion, we cultured cells from noninfected and infected mice in the presence and absence of soluble tachyzoite antigen (STAg). MLN cells isolated from noninfected mice secreted minimal amounts of IFN-γ, whether or not STAg was present (**Fig 7**). Similar to our previous findings [17], large amounts of IFN-γ were produced in the absence of further in vitro stimulation using cells from infected WT mice (**Fig 7**). This response was only marginally increased by stimulation with parasite Ag. In the absence of MyD88 and αβ T cells, spontaneous IFN-γ secretion was significantly decreased but there remained a response substantially above background (**Fig 7**). Interestingly, STAg stimulation elicited only a partial increase in IFN-γ production in MyD88 KO compared to WT cells. In cells from *Tcrb*^*-/-*^ mice there was no significant increase in production of this cytokine in response to Ag stimulation. Although it is difficult to determine in this experiment, given the bulk MLN cell population subjected to stimulation, the results suggest that in vitro STAg addition may mainly target IFN-γ by T cells, at least in part through MyD88 signaling. This conclusion is in line with findings of others that examined T cell-intrinsic MyD88 signaling during *Toxoplasma* infection [39].

## Discussion

In this study we employed *T. gondii* to examine ILC function in the MLN, the site of adaptive immune deployment during gastrointestinal infection. We found a predominance of ILC1, as well as ILC2 and a population of putative ILC that failed to express transcription factors associated with ILC1, ILC2 and ILC3 subsets. The predominance of ILC1, a result previously seen at other anatomical locations [15,16,32–34,36,40], aligns with the Type 1 cytokine-inducing properties of this pathogen. Expression of IFN-γ by ILC1 was only partially dependent upon the TLR/IL-1R signaling adaptor MyD88. ILC1 were influenced by the αβ T cell compartment, insofar as they were decreased in frequency and IFN-γ expression in *Tcrb*^*-/-*^ mice. The MLN ILC1 were highly proliferative as defined by expression of the nuclear marker Ki67. There was a slight decrease in proliferative potential in the absence of MyD88 and T cells, although the latter was not statistically significant. Regardless, high Ki67 expression in MLN ILC1 contrasts strikingly with LP ILC, where expression of this proliferation marker was uniformly only 1–5% [15].

The MyD88 molecule has been extensively studied in infection models and is known for its importance in TLR-dependent pathogen recognition and downstream signaling initiated by IL-1 family cytokines. However, the function of MyD88 extends beyond sensing and transducing signals downstream of IL-1-related receptors. For example, this molecule influences bone marrow hematopoiesis in myeloid and lymphoid compartments under steady-state conditions and during the response to infection [41,42]. It has also been found that TLR signaling through MyD88 affects neural stem cell differentiation and proliferation, and well as having an impact of mesenchymal stem cell function [43,44]. These observations require consideration when employing MyD88 global knockout mice in studies of infection.

Discriminating ILC1 from NK cells has been a challenge due to overlapping differentiation pathways, shared transcription factors and similar surface marker expression [45]. NK cells are well known to possess cytotoxic function, but this does not appear to be a major role for ILC1 [46,47]. Conversely, ILC1 are believed to produce large amounts of IFN-γ, and the cells are a significant source of innate IFN-γ during *T. gondii* infection [21,33]. In our gating strategy, we eliminated cells expressing CD11b. Expression of this integrin is a property of mature NK cells, arguing against NK cells as a major component of our ILC1 population [48]. Nevertheless, we cannot completely rule out the possibility of NK cells in the subset we identify as ILC1 because immature NK cells express only low levels of CD11b. Further complicating the issue, it was reported that *Toxoplasma* reprograms NK cells into ILC1-like cells during infection [34].

We detected a population of CD45^+^Lin^-^CD90^+^ NP cells that were negative for T-bet, RORγt and GATA3 and therefore could not be assigned ILC1, ILC2 or ILC3 identity. Along similar lines, we previously observed a population of T-bet^+^RORγt^+^ ILC1/3 cells in the LP compartment during infection, although they were absent in the MLN in the present study [16]. The NP cells observed here produced low amounts of MyD88-dependent IFN-γ, and approximately 50% expressed MyD88-dependent MHC class II molecules. The nature of these NP cells is presently unknown, but ILC possess remarkable plasticity, enabling phenotypic shifts in response to environmental cues [25]. Possibly the NP cells reflect a population of ILC in transition from one subset to another.

Another possibility is that the NP population is composed of IL-10-secreting regulatory ILC (ILCreg) [23]. Production of IL-10 is an important factor in the response to *Toxoplasma*, preventing emergence of proinflammatory tissue pathology that would otherwise result in early host lethality [49–52]. ILCreg, which are hypothesized to be the ILC counterpart to Treg T lymphocytes, are thought not to express T-bet, GATA3 or RORγt, instead relying on transcription factor Id3 for their development. There is evidence that they play a regulatory role during intestinal inflammation [24]. Nevertheless, whether regulatory ILC exist as a distinct lineage is controversial, and some studies support that ILC2 may fulfill a similar regulatory function through IL-10 production [53,54].

There are multiple lines of evidence for crosstalk between ILC and T lymphocytes that regulates function of the latter [55,56]. For example, interactions between MHCII expressing ILC2 and CD4^+^ T cells promote Type II immunity in the intestine enabling effective elimination of *Nippostrongylus brasiliensis* [27]. RORγt-expressing ILC express MHCII and can process and present antigen. Their role in the gut is to limit CD4^+^ T cell responses to the intestinal microbiota [57,58]. Here, we found that ILC2, and in particular NP cells, expressed MHCII. Interestingly, while ILC1 displayed only low levels of MHCII, in the absence of MyD88 signaling there was an increase in expression at the expense of expression by NP cells. We also note that in *Tcrb*^*-/-*^ mice there was a similar increase in ILC1 expression of MHCII.

Conversely, T cells themselves are known to regulate ILC populations. These interactions are complex, inasmuch as it has been found that T lymphocytes orchestrate ILC subsets in multiple ways dependent upon tissue location and ILC subset [59]. The absence of CD4^+^ T cells results in constitutive STAT3 phosphorylation in intestinal ILC3 and epithelial cells that is dependent upon IL-22 and IL-23 [60]. Another recent study revealed that CD4^+^ T lymphocytes regulate IL-22-producing intestinal ILC2 [61]. We also found evidence for a T cell signature on ILC distribution and function in the MLN. In *Tcrb*^*-/-*^ mice, we found an increase in ILC2 concomitant with a decrease in ILC1. There was also an increase in MHCII expression by ILC2 and ILC1. Elevated MHCII levels in ILC1 is of interest because these cells are not usually associated with expression of this molecule. It was not unexpected that IFN-γ levels were decreased in *Tcrb*^*-/-*^ mice, given the long-recognized ability of *Toxoplasma* to stimulate strong T cell dependent production of this cytokine [5,62]. Thus, it seems highly plausible that T cells imprint their influence on the ILC compartment in part or in whole through secretion of this proinflammatory cytokine.

ILC populations are rare, and there is evidence that they are redundant in the presence of an intact T cell compartment [63,64]. Nevertheless, as pointed out by others [59], there are clinical situations, including infection and cancer, where immunodeficiency could be expected to elevate the predominance of ILC interactions in optimizing immunity. Understanding ILC function in the presence of defective innate immune signaling and absent adaptive immune responses is therefore an important area of further continued investigation.

## Materials and methods

### Ethics statement

Experiments were performed in accordance with the National Institutes of Health Guide for the Care and Use of Laboratory Animals (8^th^ edition). Protocols were approved by the Institutional Animal Care and Use Committee at the University of New Mexico (Animal Welfare Assurance Number A4023-01). All efforts were made to minimize the number of experimental animals, pain, and distress throughout these studies.

### Mice

C57BL/6J (Cat. 000664), Myd88tm1.1Defr/J (Cat. 009088; *Myd88*^*-/-*^), and Tcrbtm1Mom/J (Cat. 002118; *Tcrb*^*-/-*^) mice were purchased from The Jackson Laboratory (Bar Harbor, ME). Colonies were maintained in the University of New Mexico Department of Biology Animal Research Facility for experimental use. Strains CBA/J (Cat. 000656) and J:ARC(S) (Cat. 034608; Swiss Webster) mice were obtained from The Jackson Laboratory, housed in the same facility, and used to maintain *T. gondii* cysts. Mice were used at 6–12 weeks of age. Animals were euthanized by CO_2_ asphyxiation followed by secondary cervical dislocation. Anesthesia was accomplished using isoflurane. A four-point scoring system was employed to determine mouse endpoints [65].

### Parasites and infections

Cysts of the Type II ME49 *T. gondii* strain were maintained by i. p. passage of infected brain homogenate every 4–8 weeks. For oral inoculation, brain tissue from chronically infected mice was homogenized in phosphate-buffered saline (PBS), cysts enumerated, then the brain homogenate was administered to mice (30 cysts in 200 μl) by gavage with an 18 Ga blunt-ended needle.

### Isolation of immune cells

MLN were collected in Hank’s Balanced Salt Solution (HBSS) supplemented with 1% bovine growth serum (HyClone, Logan, UT, Cat. SH30541.03), penicillin (100 U/ml), and streptomycin (100 μg/ml). Tissue was homogenized and filtered through a 70 μm cell strainer, then the cell suspension was washed by centrifugation (5 min, 290 x g) prior to resuspension in supplemented HBSS. LP cells were obtained from small intestine tissue. The intestinal tissue was gently flushed with PBS, Peyer’s patches were excised, and tissue was cut longitudinally into 1 cm pieces. The tissue pieces were placed on a rotating platform (20 min, 37°C) in HBSS supplemented with 10 μM EDTA and 1 mM dithiothreitol. Media was carefully decanted, and the procedure repeated. This was followed by incubation in complete Dulbecco’s Modified Eagle’s medium (cDMEM) composed of DMEM (Corning Inc., Corning, NY, Cat. 10–013-CVR) supplemented with 1% bovine growth serum (Hyclone), MEM non-essential amino acids (Thermo Fisher Scientific, Waltham, MA, Cat. 11140050), penicillin (100 U/ml) and streptomycin (100 μg/ml), HEPES buffer (Thermo Fisher Scientific, Cat. 15630080) 2-mercaptoethanol (30 mM; Sigma Aldrich Corporation, St. Louis, MO, Cat. M3148). In addition, collagenase (300 U/ml; Worthington Biochemical Corporation, Lakewood, NJ, Cat. LS004197) was added to the cDMEM and the suspension was incubated for 1 hr on a rotating platform at 37°C for cell dissociation. The resulting cell suspension was filtered through a 70 μm strainer then subjected to centrifugation (500 x g, 20 min, 20°C, no terminal brake) on a discontinuous gradient consisting of 40% and 80% Percoll (Sigma-Aldrich Cat. P4937) diluted in cDMEM. Cells at the 40/80% interphase were collected, washed twice in DMEM (400 x g, 5 minutes, 10°C) and used in downstream assays. Splenocytes were isolated by macerating spleens and filtering through a 70 μm filter with PBS. Cells were briefly lysed with 2 ml ACK buffer (Thermo Fisher Scientific, Cat. A1049201) and rinsed with PBS. Cell viabilities were routinely 80–90%.

### Cell culture and ELISA

10^6^ MLN cells/ml were cultured ex vivo in cDMEM alone or with soluble tachyzoite antigen (STAg; 50 μg/ml) prepared as described [16]. Supernatants were harvested after 72 hr of incubation (37°C, 5% CO_2_) and either stored at -80°C or used immediately for cytokine measurement. IFN-γ was analyzed by ELISA following the manufacturer’s instructions (Thermo Fisher Scientific, Cat. 88-7314-88).

### Flow cytometry

Cells were washed in PBS prior to staining for flow cytometry. Then 10^6^ cells were incubated with Zombie Aqua Fixable Viability dye (Cat. 423102; BioLegend, San Diego, CA) at a dilution of 1:1000 for 5 min in PBS to stain dead cells. Cells were subsequently incubated with fluorochrome-labelled antibodies at a 1:100 dilution in FACS buffer consisting of 1% bovine growth serum with 30 mM NaN_3_ in PBS (20 minutes, 4°C). Fluorochrome-labelled antibodies to surface antigens included Brilliant Violet 570 rat anti-mouse CD45 (BioLegend, Cat. 103136), anti–lineage 1 mixture consisting of PerCP/Cy5.5 rat anti-mouse CD8α (BioLegend, Cat. 100734), PerCP/Cy5.5 rat anti-mouse CD5 (BioLegend, Cat. 100624), PerCP/Cy5.5 Armenian Hamster anti-mouse CD3ε (BioLegend, Cat. 100328), anti–lineage 2 mixture composed of APC-eFluor 780 rat anti-mouse CD11b (Thermo Fisher Scientific, Cat. 47-0112-82), APC-eFluor 780 Armenian Hamster anti-mouse CD11c (Thermo Fisher Scientific, Cat. 47-0114-82), APC-eFluor 780 rat anti-mouse B220 (Thermo Fisher Scientific, Cat. 47-0452-82), PE rat anti-mouse CD90.2 (BioLegend, Cat. 140308), and PE/Cyanine7 anti-mouse I-A/I-E (BioLegend, Cat. 107630). After washing cells, the samples were fixed and permeabilized using a commercially obtained fixation/permeabilization solution (Thermo Fisher Scientific, Cat. 00-5523-00) following manufacturer instructions. Staining for intracellular markers was then accomplished by incubation of cells with fluorochrome-labelled antibodies in permeabilization buffer (Thermo Fisher Scientific, Cat. 00-5523-00) for 1 hr at 4°C. Antibodies to intracellular antigens included Alexa Flour 647 mouse anti-mouse T-bet (BioLegend, Cat. 644804), Brilliant Violet 650 mouse anti-mouse RORγt (BD Biosciences, Franklin Lakes, NJ; Cat. 564722), Brilliant Violet 421 mouse anti-mouse GATA3 (BioLegend, Cat. 653814), PE/Cyanine7 rat anti-mouse IFN-γ (BioLegend, Cat. 505826), and FITC rat anti-mouse Ki67 (BioLegend, Cat. 151212). Antibodies were used at a dilution of 1:100. After staining, samples were washed and resuspended in FACS buffer. For IFN-γ expression studies, prior to antibody labelling, 10^6^ cells were incubated in cDMEM alone or stimulated with phorbol-12-myristate-13-acetate (3 μM; Sigma-Aldrich Corporation, Cat. 524400), ionomycin (2 μM; Thermo Fisher Scientific, Cat. J62448.MCR), and brefeldin A (36 μM; BioLegend, Cat. 420601) for 4 hr at 37°C. Flow cytometry was performed on an Attune NxT 4 laser flow cytometer (Thermo Fisher Scientific). Data were analyzed employing FlowJo v.10.10 software (BD Biosciences).

### *In vivo* cytokine depletion

Mice received 0.5 mg monoclonal rat anti-mouse IL-12p40 (BioXCell, Lebanon, NH, Cat. BE0051) or 0.5 mg Rat IgG2a Isotype control (Jackson ImmunoResearch, Cat. 012-000-002) in 200 μl PBS via intraperitoneal injection. Treatment began one day prior to oral infection, and subsequently repeated on Days 1, 3, 6 and 8 post infection.

### Statistical analyses

Prism software (GraphPad, La Jolla, CA) was used for graphing and statistical analyses. Datasets were compared using multiple unpaired two-tailed Student t tests or One-way ANOVA with Tukey’s multiple comparison test. A significant threshold of 95% confidence interval was used to identify meaningful differences among the groups. All experiments were biologically replicated two or more times.

## Supporting information

S1 Fig RORγt expression in thymocytes. (A) Representative scatterplot of CD4 and CD8 expression by C57BL/6 thymocytes. Expression of RORγt in CD4^+^ (B) and DP (C) cells. (D and E) Percentage values for individual mice expressing CD4 and CD8 (D) and RORγt expression in CD4 and DP cells (E). DP, double-positive for CD4 and CD8.
(TIF)

S2 Fig Distribution of ILC subsets in noninfected tissues. Representative scattergrams of noninfected C57BL/6 mice ILC subset distribution in MLN (**A**), LP (**C**), and spleen (**E**). Numbers indicate percentages falling within the indicated quadrants. Collective percentages of n=5 mice in MLN (**B**), LP (**D**) and spleen (**F**). Each symbol represents an individual animal. Unpaired Student t test comparison to ILC1 was performed, where * p < 0.05, ** p<0.01, *** p<0.001.
(TIF)
