# Supplementary figures and images for: Influence of MyD88 and αβ T cells on mesenteric lymph node innate lymphoid cell populations during Toxoplasma gondii infection
Source: PLoS One. 2025 Apr 29;20(4):e0322116. doi: 10.1371/journal.pone.0322116 (PMC12040133; doi:10.1371/journal.pone.0322116)

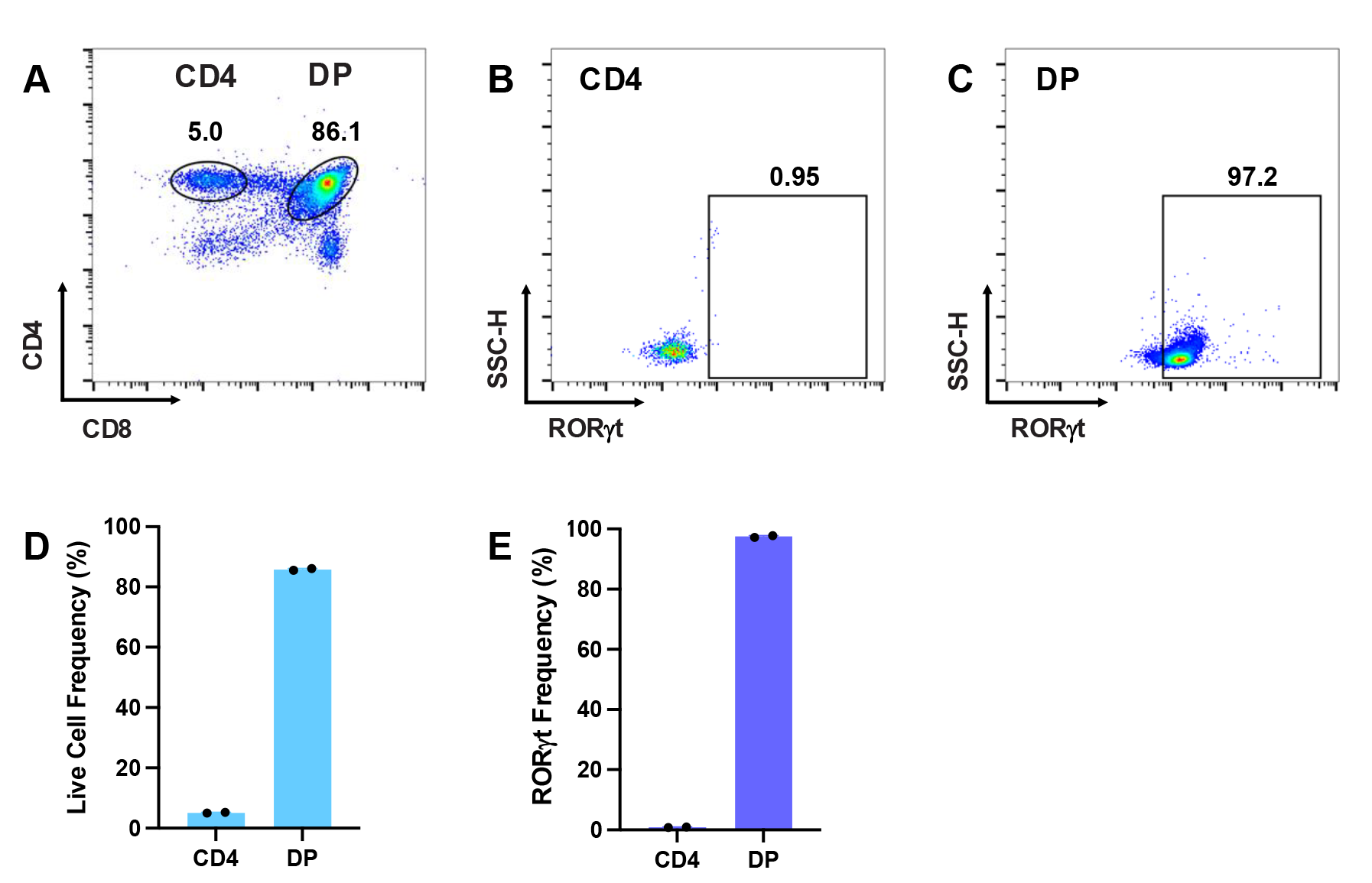

Supplement: S1 Fig — (A) Representative scatterplot of CD4 and CD8 expression by C57BL/6 thymocytes. Expression of RORγt in CD4+ (B) and DP (C) cells. (D and E) Percentage values for individual mice expressing CD4 and CD8 (D) and RORγt expression in CD4 and DP cells (E). DP, double-positive for CD4 and CD8. (TIF) [file pone.0322116.s001.tif]

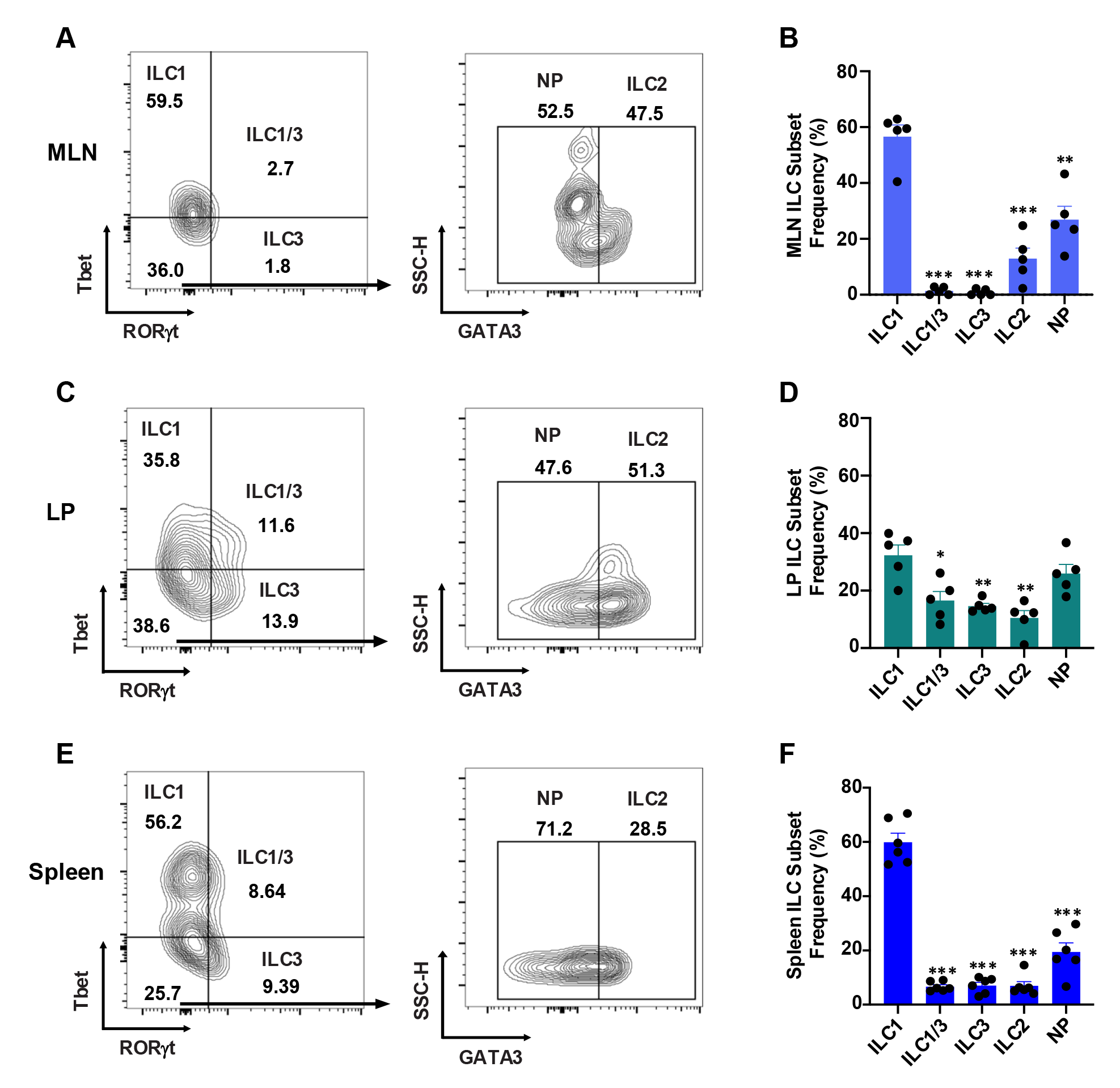

Supplement: S2 Fig — Representative scattergrams of noninfected C57BL/6 mice ILC subset distribution in MLN (A), LP (C), and spleen (E). Numbers indicate percentages falling within the indicated quadrants. Collective percentages of n=5 mice in MLN (B), LP (D) and spleen (F). Each symbol represents an individual animal. Unpaired Student t test comparison to ILC1 was performed, where * p < 0.05, ** p<0.01, *** p<0.001. (TIF) [file pone.0322116.s002.tif]
